# Supplementary material for: Globally invariant metabolism but density-diversity mismatch in springtails
Source: Nat Commun. 2023 Feb 7;14:674. doi: 10.1038/s41467-023-36216-6 (PMC9905565; doi:10.1038/s41467-023-36216-6)
Supplement: Supplementary file 1 — Supplementary Information [file 41467_2023_36216_MOESM1_ESM.pdf]

Globally invariant metabolism but density-diversity mismatch in springtails

Supplementary information and materials

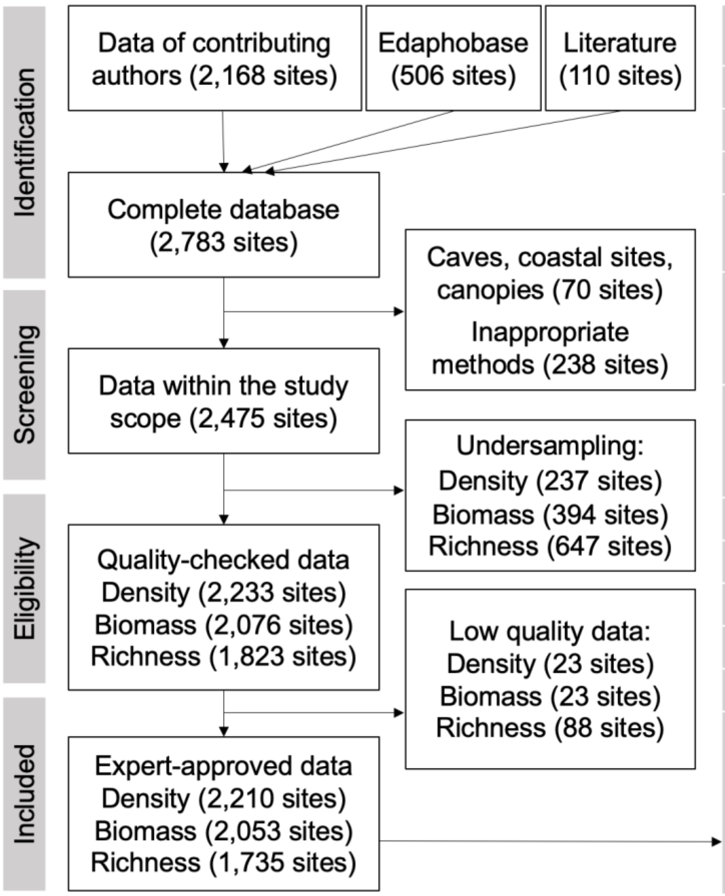

Major data providers		
	Sites	Samples
Edaphobase	488	77
Anatoly Babenko	332	2,667
Sophie Joimel, Angélique Renaud, Céline Pernin and Jérôme Cortet	139	4,407
Ľubomír Kováč, Natália Raschmanová and Peter Čuchta (collaborator: Juha Alatalo)	136	3,239
Natalia Kuznetsova (collaborators: Mikhail Potapov, Anna Bokova, Anna Saraeva)	115	10,507
Stef Bokhorst	84	893
Daniel Winkler	84	420
Pascal Querner	63	782
Jean-François Ponge	32	1,118
Viesturs Melecis and Edite Jucevica	1	3,300
Anton Potapov (collaborators: Anastasiya Stryuchkova, Stefan Scheu, Winda Ika Susanti) includes literature data	176	1,955

Supplementary Fig. 1 | Flow diagram of data compilation and selection. Major data providers of #GlobalCollembola whose data were used in the analysis are given in the shaded table on the right side. Providers are ordered based on the number of sites, but exemplar datasets with extensive sampling efforts (number of samples) are given to illustrate the available data.

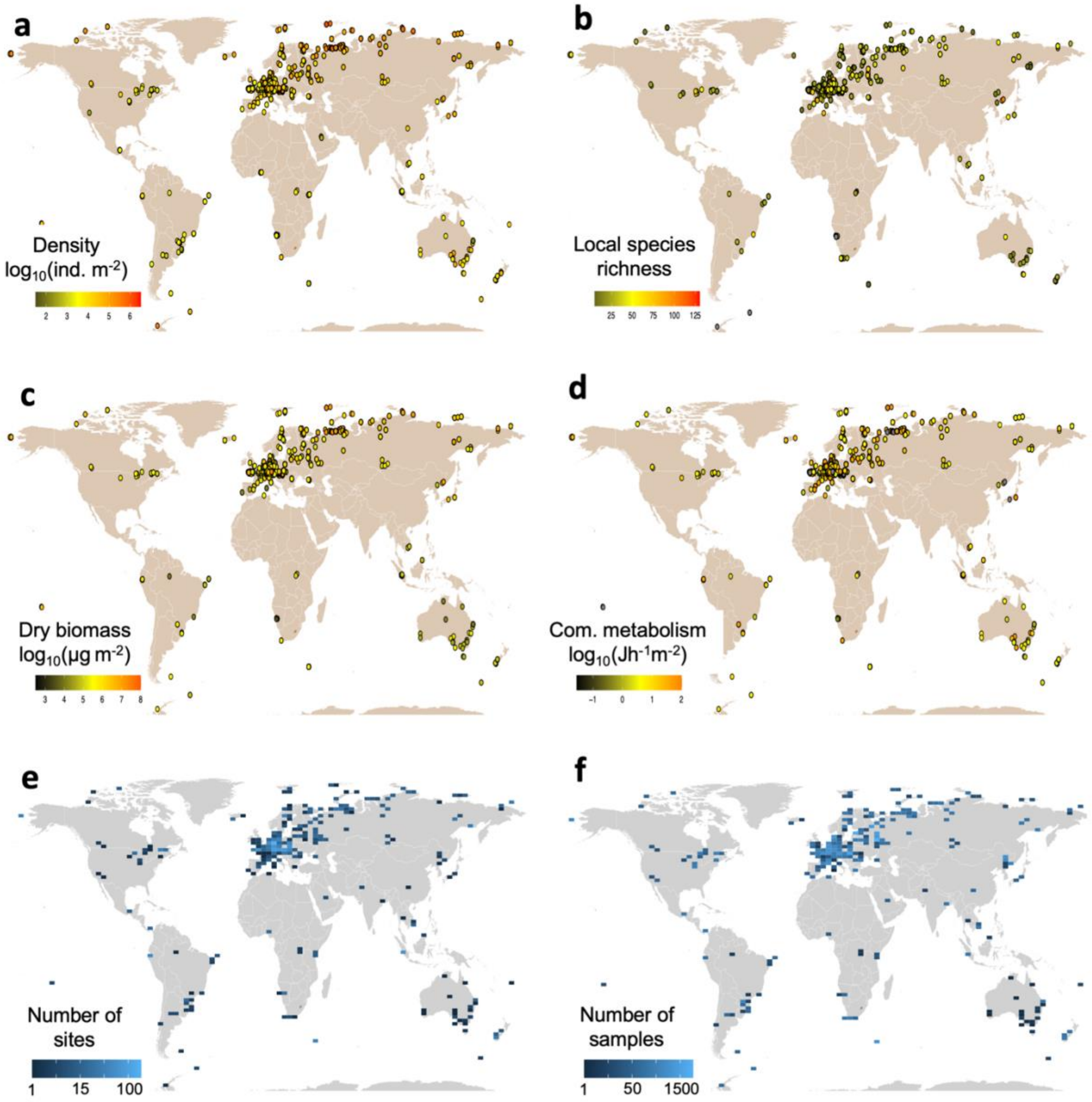

Supplementary Fig. 2 | Selected sampling sites that were used in the analysis and the global sampling effort. **A**, Density (n = 2210), **b**, Local species richness (n = 1735); **c**, Dry biomass (n = 2053); **d**, Community metabolism (n = 2053). Data scales are logarithmic except for local species richness. Global sampling effort represented in the total number of sites (e) and samples (f) in each latitudinal-longitudinal bin (100 x 100 bins in total; not the logarithmic scales). Source data are provided as a Source Data file.

Supplementary Table 1 | Regression coefficients used to estimate the dry and fresh body masses of springtail genera based on body lengths. For each genus, the average body mass (*M*) [μg dry weight] was calculated from the average body length (*L*) [mm] using the power equation: $M = a*L\^b$, where *a* is the normalisation coefficient and *b* is the exponent. Abdomen length of Symphypleona was used in the original equations and was assumed to be 0.83 of the total body length. Two sets of coefficients coming from two independent studies[56,57] were used for each morphogroup (a_1, b_1 and a_2, b_2) and the two estimates of dry body mass were averaged. Fresh body mass was calculated from the resulting average by dividing it by the proportion of the dry weight.

Morphogroup	Normalisation (a_1)	Exponent (b_1)	Normalisation (a_2)	Exponent (b_2)	Dry weight proportion
Entomobryoidea	11.749 ± 1.060	2.52 ± 0.10	14.256 ± 1.031	2.708 ± 0.061	0.30
Isotomidae (<1.5 mm)	6.457 ± 1.140	2.99 ± 0.12	5.623 ± 1.037	2.799 ± 0.136	0.36
Isotomidae (≥1.5 mm)	5.623 ± 1.250	3.28 ± 0.30	8.427 ± 1.080	3.223 ± 0.253	0.36
Onychiuridae	4.266 ± 1.090	2.75 ± 0.10	5.598 ± 1.076	2.769 ± 0.196	0.30
Poduromorpha (excl. Onychiuridae)	9.772 ± 1.220	2.55 ± 0.25	5.598 ± 1.076	2.769 ± 0.196	0.30
Symphypleona. (*sensu lato*)	190.546 ± 1.000	3.627 ± 0.143	39.628 ± 1.213	3.796 ± 0.837	0.21
Tomoceridae	9.204 ± 1.040	2.744 ± 0.048	14.256 ± 1.031	2.708 ± 0.061	0.25

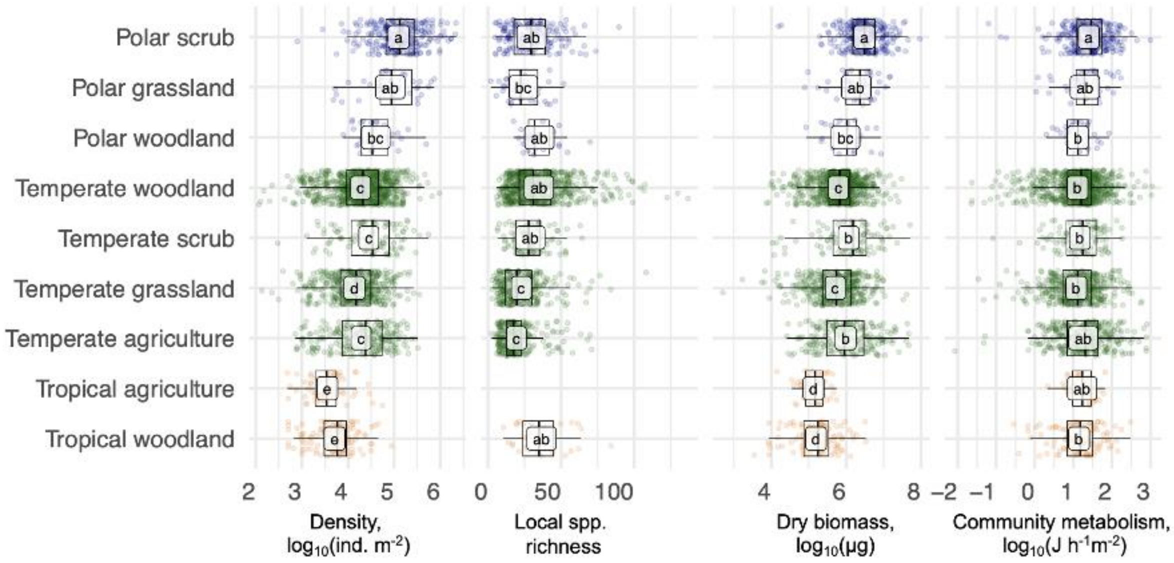

Supplementary Fig. 3 | Mean estimates for community parameters in different ecosystem types. Points represent sites, labels represent mean values, means sharing the same letter are not significantly different (Tukey's HSD one-sided test for multiple comparisons[64]). For ecosystem classification see Methods. Standard box and whiskers plots were used to display the data (median, quartiles and maximum/minimum values of the data that is within 1.5 times the interquartile range): polar scrub (n = 253 independent sites), polar grassland (n = 39), polar woodland (n = 28), temperate woodland (n = 907), temperate scrub (n = 104), temperate grassland (n = 445), temperate agriculture (n = 374), tropical agriculture (n = 68) and tropical forest (n = 141). Source data are provided as a Source Data file.

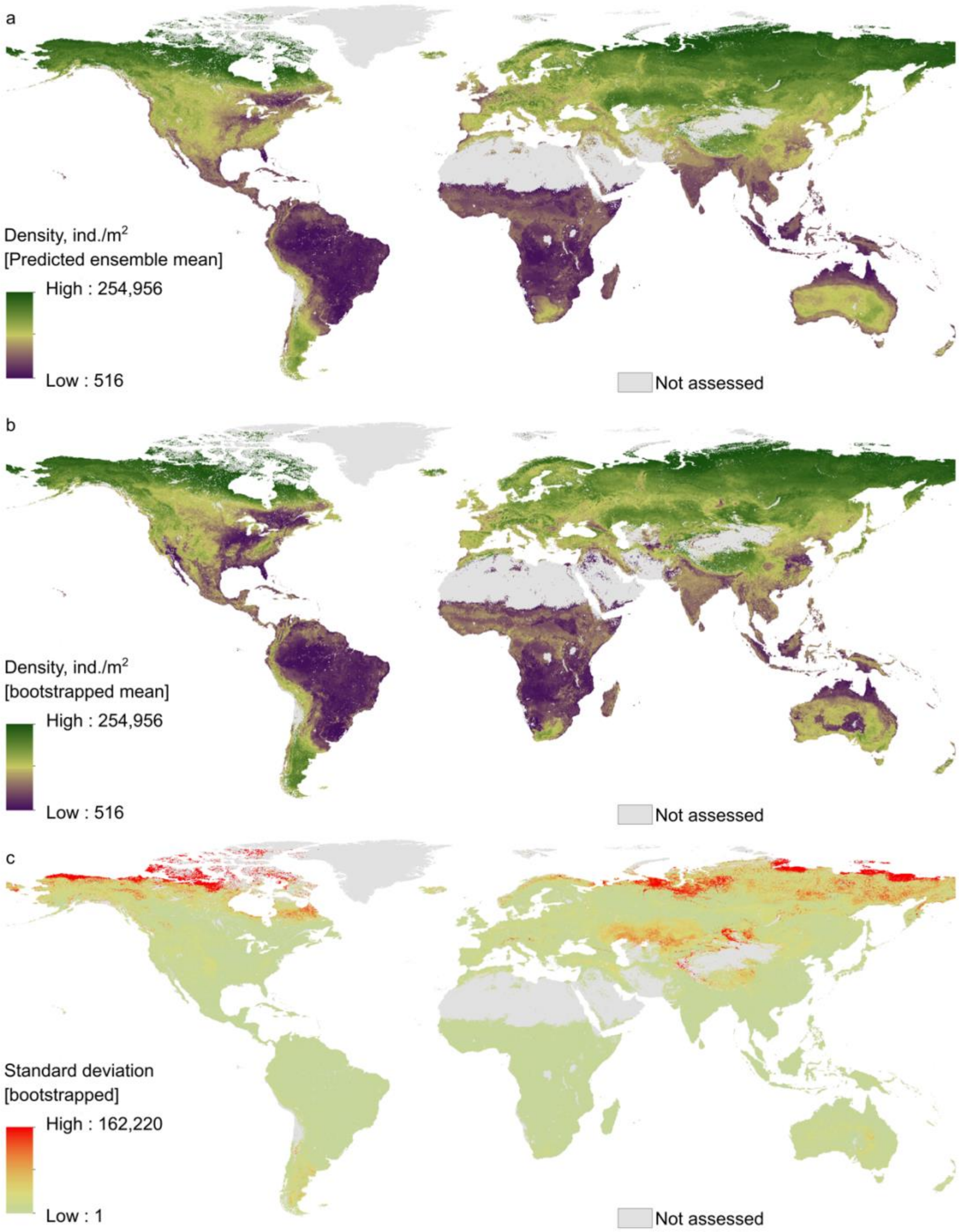

Supplementary Fig. 4 | Global unmasked projection of springtail density. Distribution was predicted with the random forest algorithm (a) based on the entire dataset and (b) using mean prediction after bootstrapping data by continents ($R^2 = 0.57 \pm 0.04$). Green colour identifies hot spots, violet colour cold spots. The bottom map (c) shows the standard deviation across the bootstrapped predictions (red – high, yellow – low). All data were projected at the 30 arcsec (approximately 1 km^2) pixel scale.

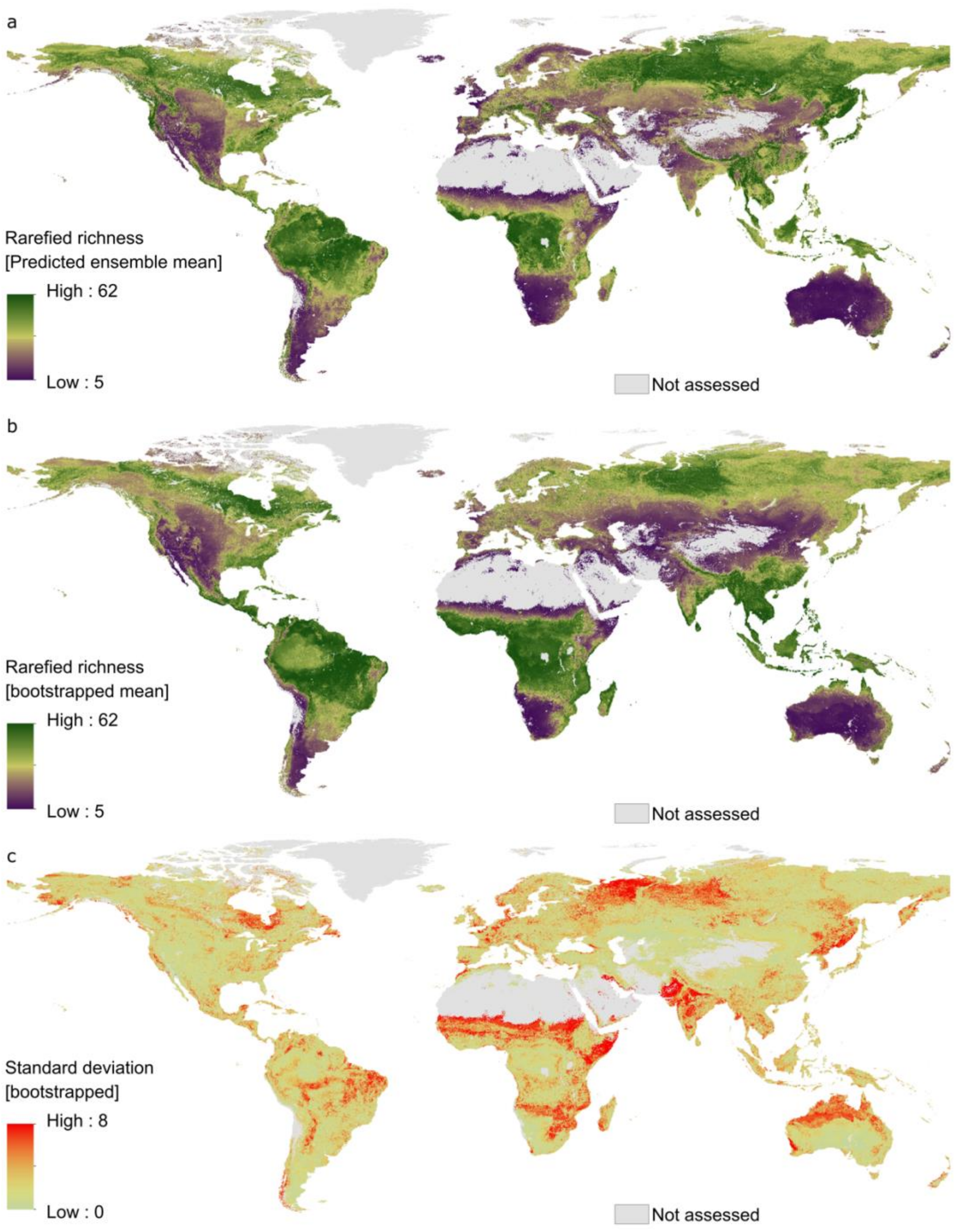

Supplementary Fig. 5 | Global unmasked projection of springtail local species richness. Distribution was predicted with the random forest algorithm (a) based on the entire dataset and (b) using mean prediction after bootstrapping data by continents ($R^2 = 0.31 \pm 0.06$). Green colour identifies hot spots, violet colour cold spots. The bottom map (c) shows the standard deviation across the bootstrapped predictions (red – high, yellow – low). All data were projected at the 30 arcsec (approximately 1 km^2) pixel scale.

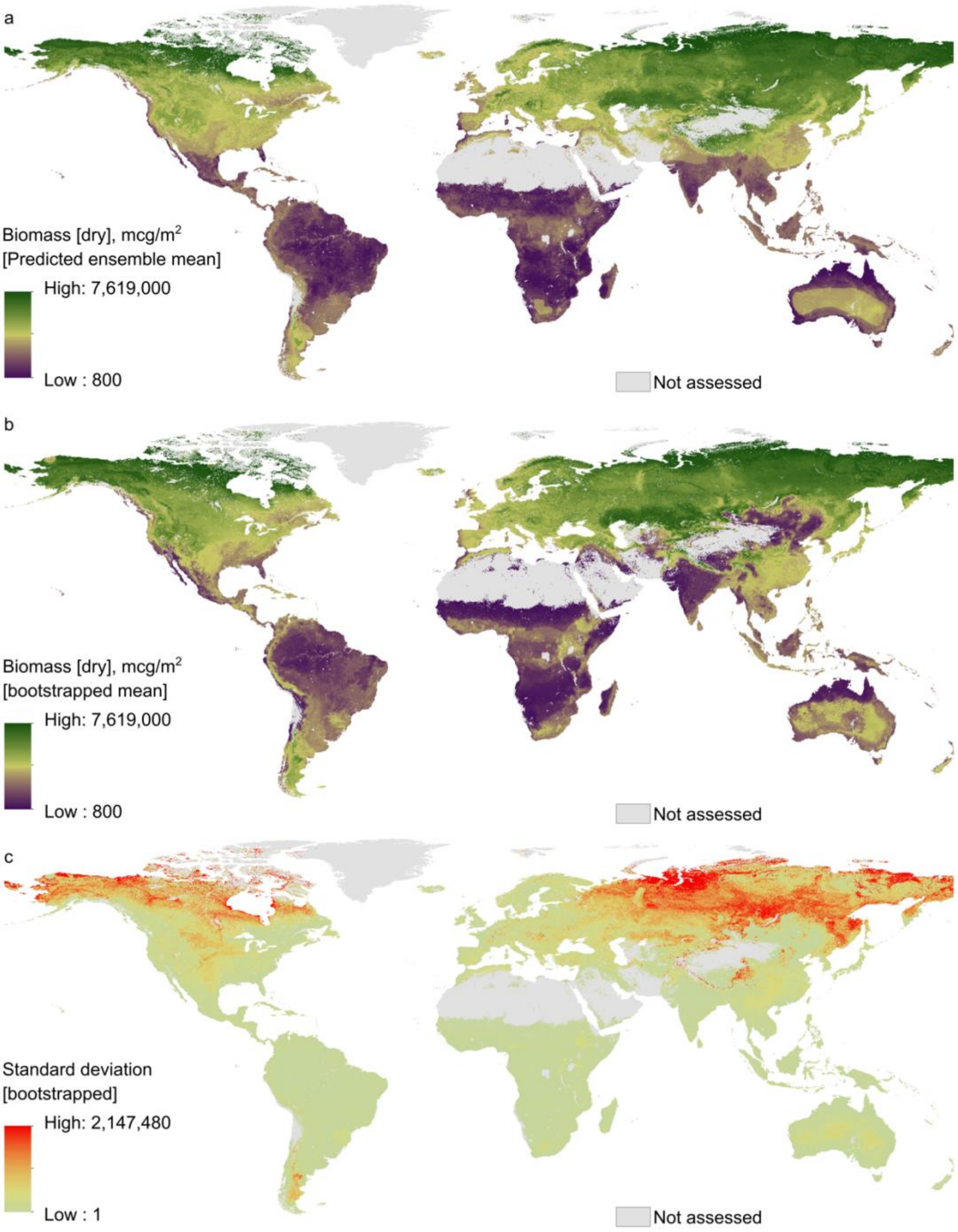

Supplementary Fig. 6 | Global unmasked projection of springtail biomass. Distribution was predicted with the random forest algorithm (a) based on the entire dataset and (b) using mean prediction after bootstrapping data by continents ($R^2 = 0.47 \pm 0.05$). Green colour identifies hot spots, violet colour cold spots. The bottom map (c) shows the standard deviation across the bootstrapped predictions (red – high, yellow – low). All data were projected at the 30 arcsec (approximately 1 km^2) pixel scale.

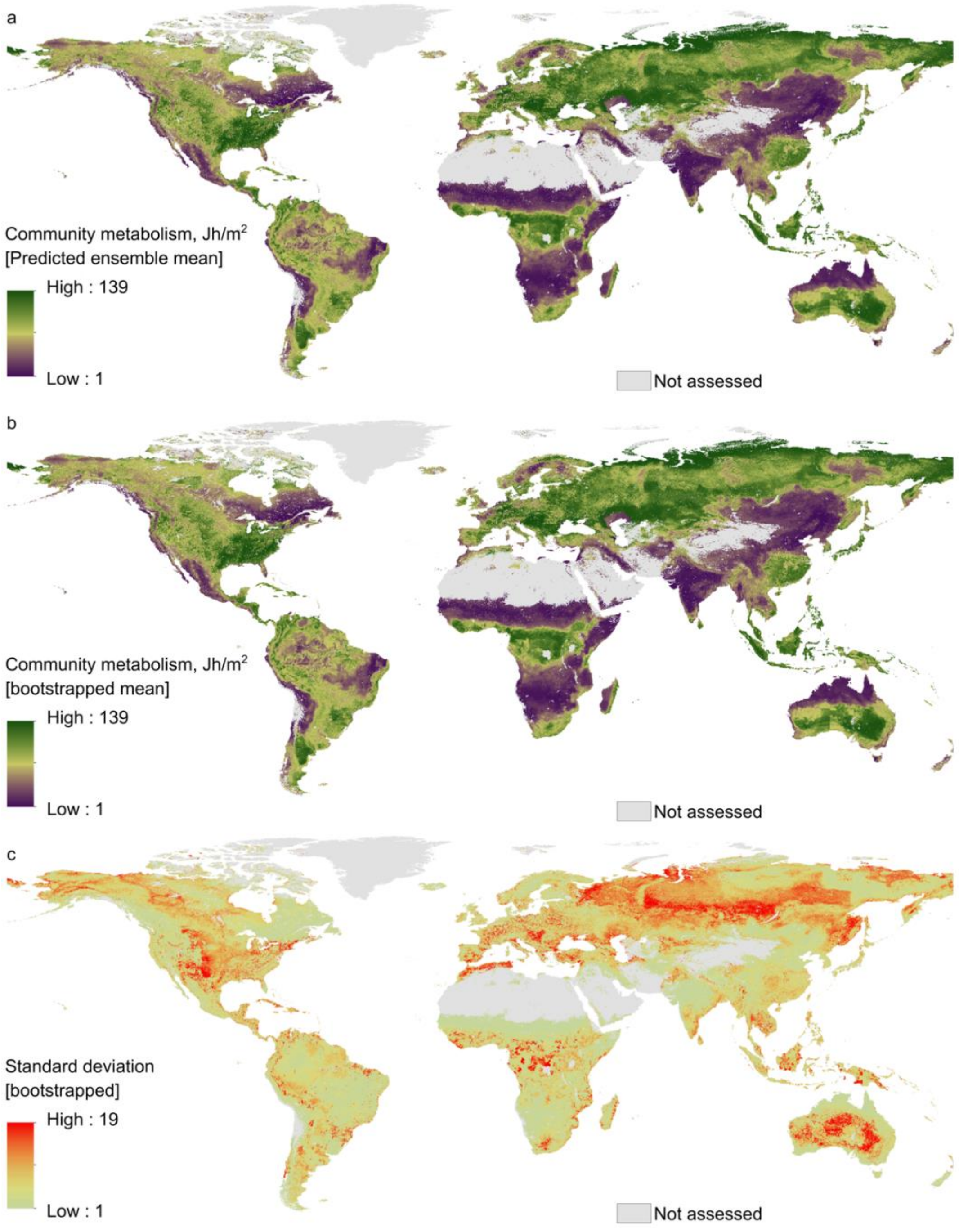

Supplementary Fig. 7 | Global unmasked projection of springtail community metabolism. Distribution was predicted with the random forest algorithm (a) based on the entire dataset and (b) using mean prediction after bootstrapping data by continents ($R^2 = 0.33 \pm 0.09$). Green colour identifies hot spots, violet colour cold spots. The bottom map (c) shows the standard deviation across the bootstrapped predictions (red – high, yellow – low). All data were projected at the 30 arcsec (approximately 1 km^2) pixel scale.

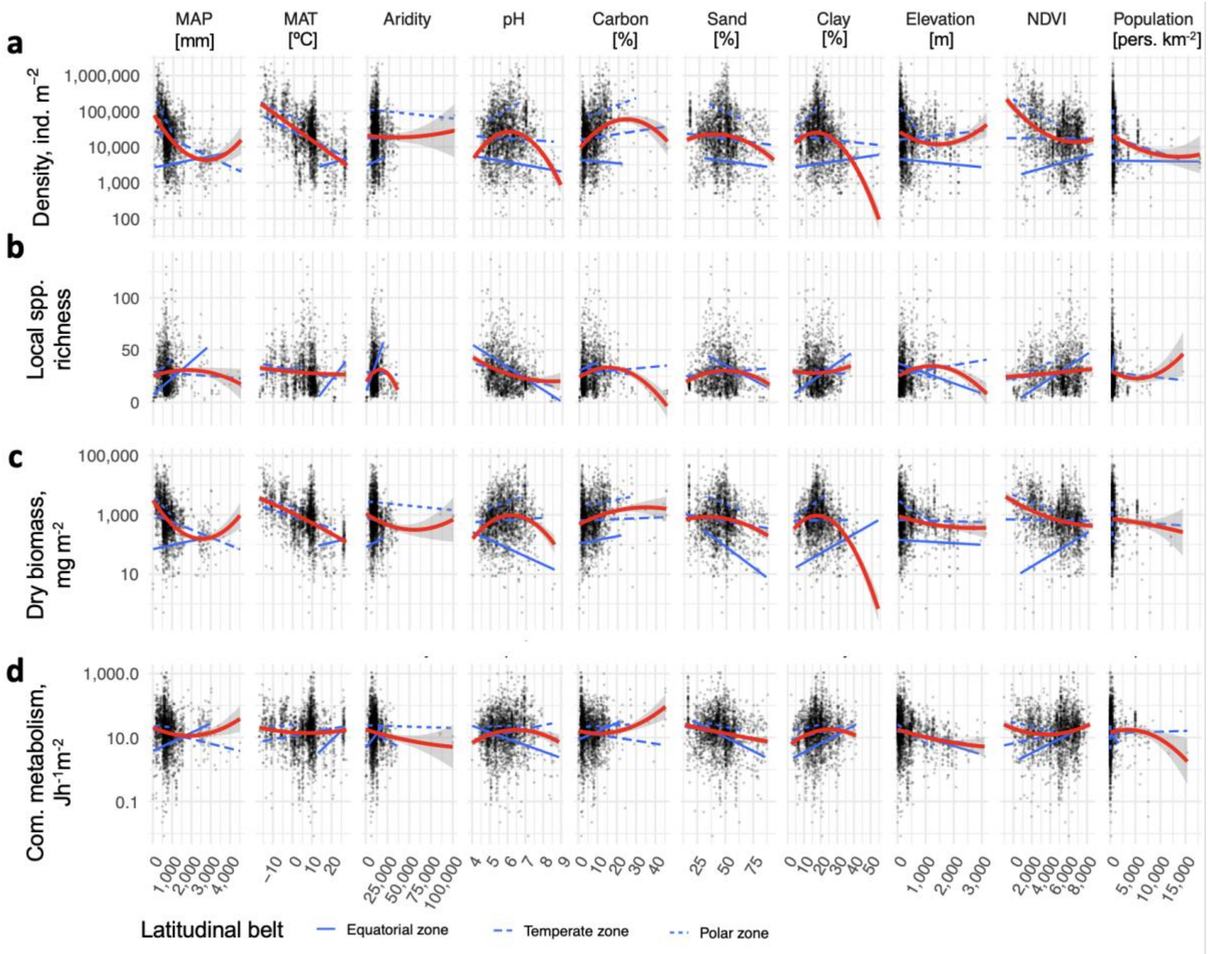

Supplementary Fig. 8 | Associations of selected environmental variables with springtail density, local species richness, dry biomass and community metabolism. Quadratic function was used for approximation to illustrate global trends (red line; mean approximation and the 95% confidence interval). Blue lines show linear trends in equatorial (solid), temperate (long dash) and polar zones (short dash). Analysed variables are: density (n = 2,210 independent sites), local species richness (n = 1,735), and biomass and community metabolism (n = 2,053). Source data are provided as a Source Data file.

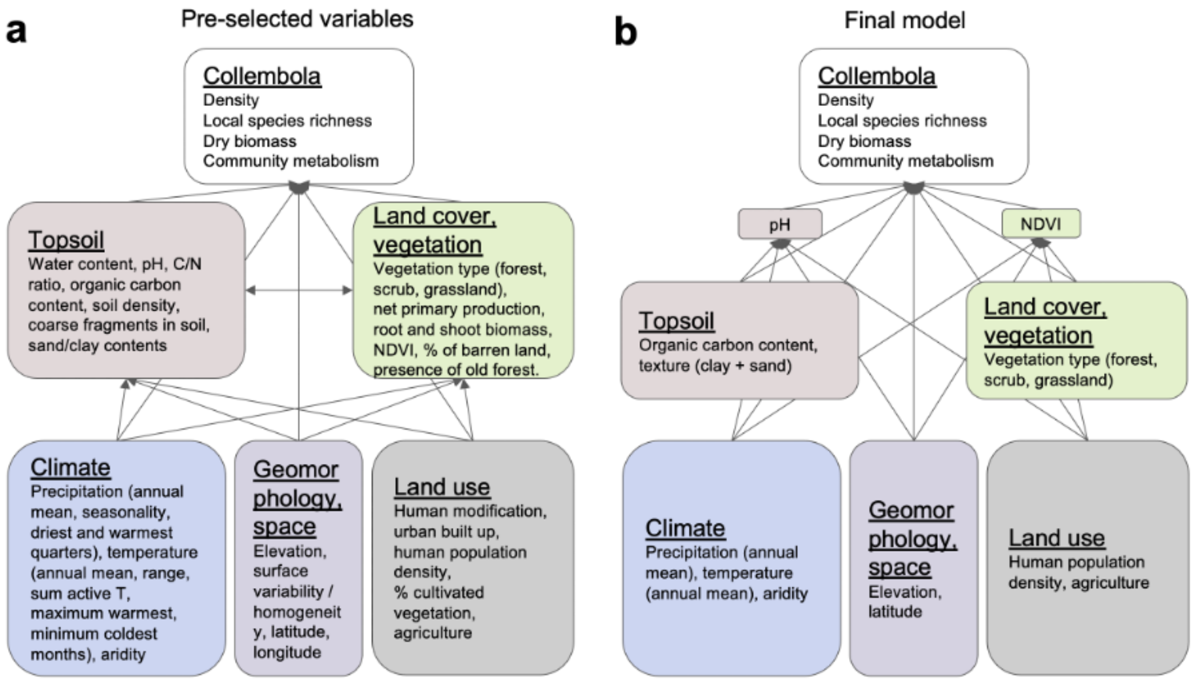

Supplementary Fig. 9 | Initial and final relationship diagram in the path analysis.

Factors directly and indirectly affecting community parameters of springtails at the global scale were pre-selected based on expert opinion (a). Factors in the final model (b) were further selected according to their global availability and collinear factors were removed. The global distributions of pH and NDVI (Normalized Difference Vegetation Index) are initially modelled based on other factors, which was accounted for in the final model.

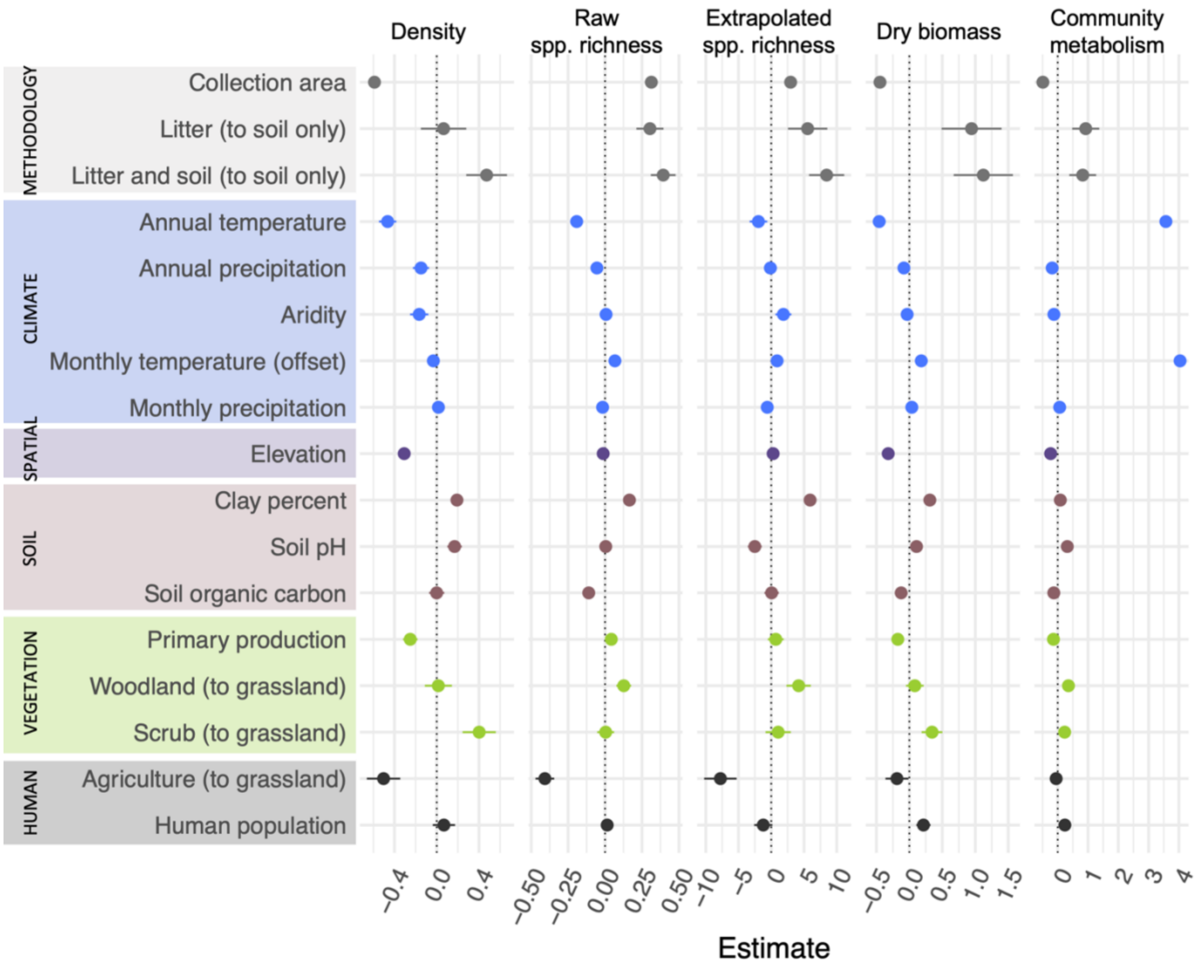

Supplementary Fig. 10 | Environmental drivers and the effect of collection methods on springtail community parameters at the global scale. Percent effect sizes (means and standard errors) from linear mixed-effects models are shown. Factors were selected to minimise collinearity. Analysis was based on sampling events (sampling site – sampling time combinations; n = 2884 for density, n = 2540 for raw species richness; n = 1708 for extrapolated species richness; n = 2462 for dry biomass; n = 2289 for community metabolism); sampling site was used as the random effect. Source data are provided as a Source Data file.

Supplementary Note: Extrapolation spatial validation

We observed some residual spatial autocorrelation in our extrapolation models at ranges below 150 km for density, below 100 km for community metabolism and below 150 km for extrapolated species richness (Fig. 1). The highest Moran's I value as 0.07 (7% correlation). With increasing buffer sizes, the coefficient of determination R^2 decreased. However, at the scales at which we observe positive autocorrelation (i.e., significant Moran's I values), R^2values remain positive (Supplementary Fig. 11). Overall, we had a fairly good predictions for density and biomass also when leaving out nearby points, up to 500 km. For extrapolated species richness and community metabolism the R^2 dropped below zero from ~200 km distances suggesting a lower reliability of our predictions.

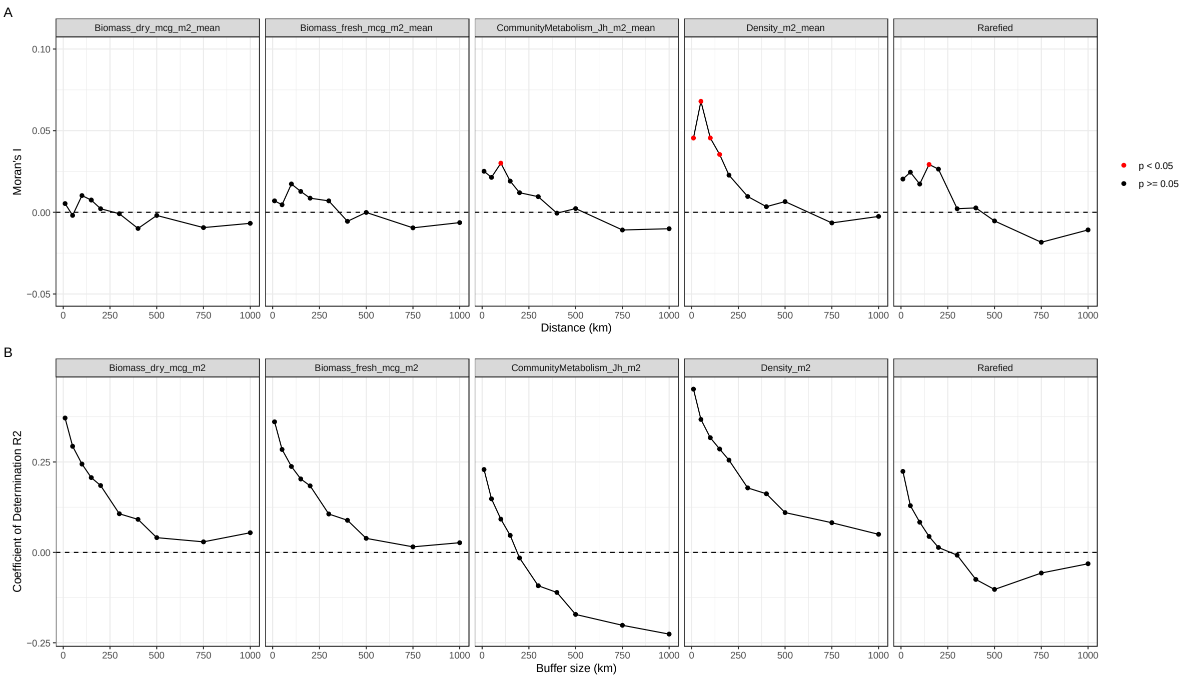

Supplementary Fig. 11 | Spatial validation of the global extrapolations. Moran's I values across varying distance thresholds (a). Points coloured in red indicate significant Moran's I values. Spatially buffered leave-one-out cross-validation tests across varying buffer sizes (b). Analysed variables are: density (n = 2,210 independent sites per model), local species richness (n = 1,735), and biomass and community metabolism (n = 2,053).

Supplementary Table 2 | The effects of sampling depth, considered microhabitats, climatic zones and habitats on the density of Collembola. Results of analysis of variance (one-tailed; n = 2,471 sites).

	Df	Sum Sq	Mean Sq	F value	p
Collection depth	1	0.6	0.6	1.5	0.2189
**Microhabitats**	**2**	**58.1**	**29.0**	**73.9**	**$7.1*10^{-32}$**
Climate zone	2	286.4	143.2	364.4	**$3.8*10^{-139}$**
Habitat	5	18.1	3.6	9.2	**$1.1*10^{-8}$**
Collection depth:Microhabitats	2	1.8	0.9	2.2	0.1076
**Collection depth:Climate zone**	**2**	**10.6**	**5.3**	**13.5**	**$1.5*10^{-6}$**
**Collection depth:Habitat**	**5**	**12.7**	**2.5**	**6.5**	**$5.6*10^{-6}$**
Microhabitats:Climate zone	4	3.2	0.8	2.0	0.0870
Microhabitats:Habitat	7	3.9	0.6	1.4	0.1918
Climate zone:Habitat	8	3.8	0.5	1.2	0.2869
Residuals	2432	955.7	0.4	NA	NA

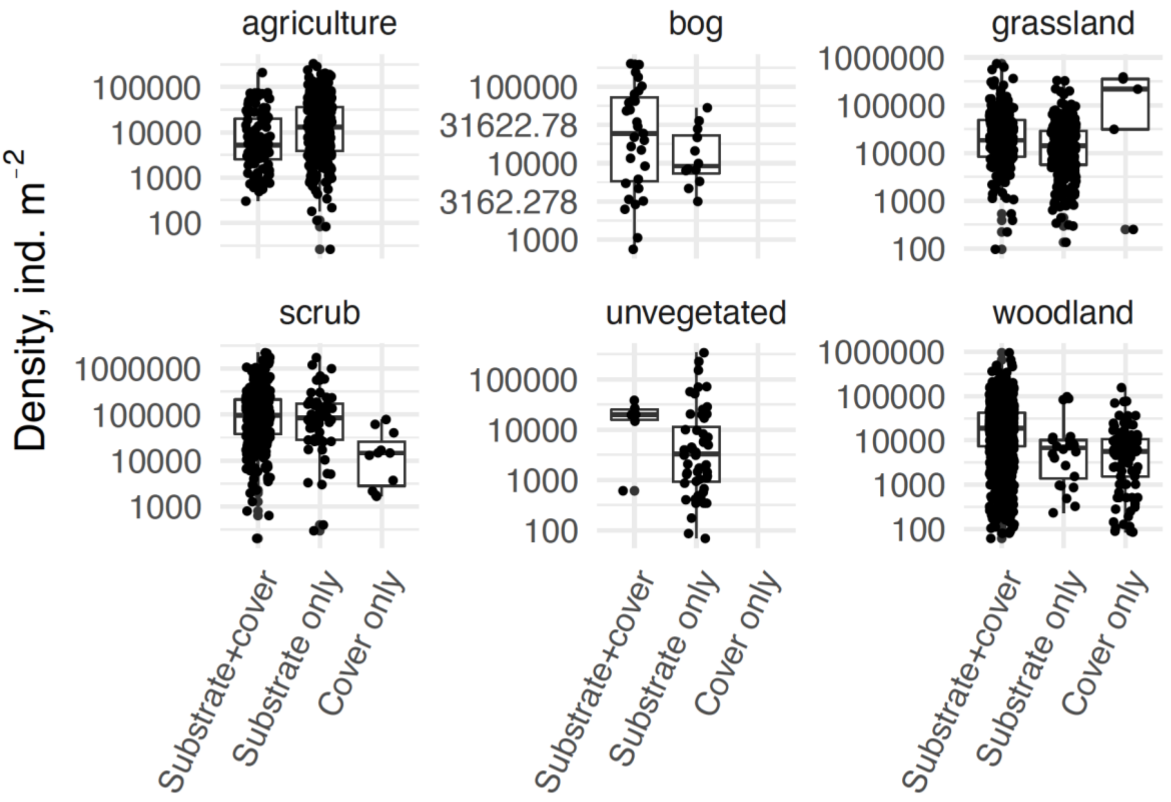

Supplementary Fig. 12 | Density of Collembola across habitats and microhabitats sampled. Note the logarithmic y axis scale. Standard box and whiskers plots were used to display the data (median, quartiles and maximum/minimum values of the data that is within 1.5 times the interquartile range; total n = 2,471 sites). Source data are provided as a Source Data file.

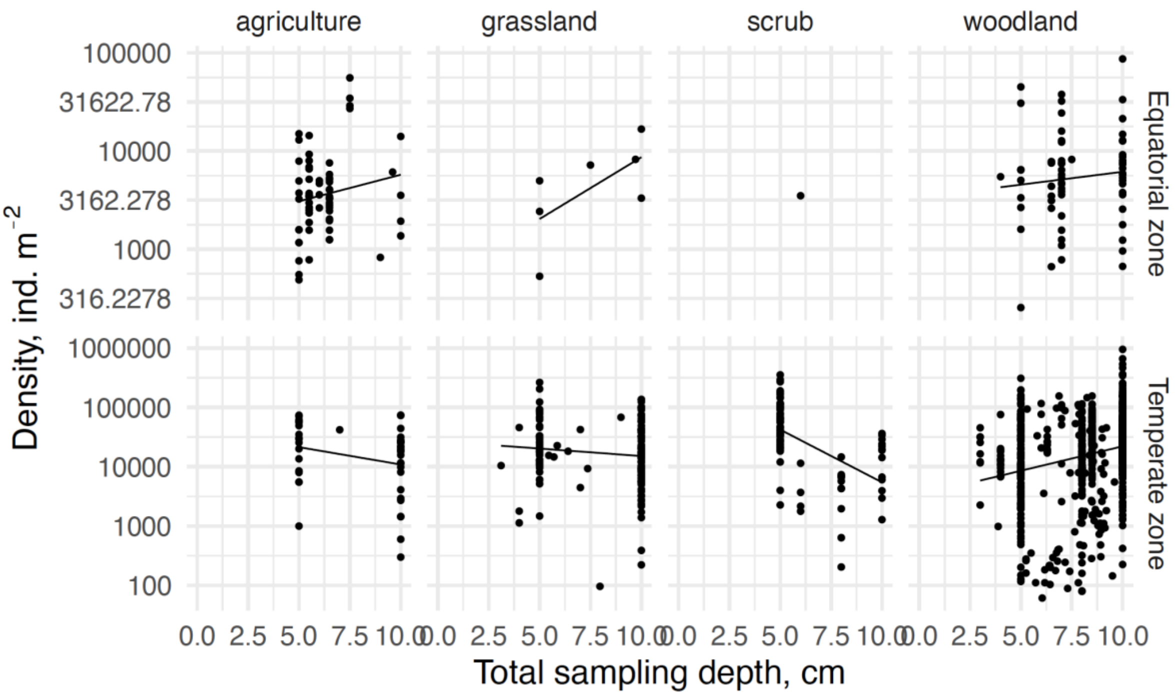

Supplementary Fig. 13 | Density of Collembola depending on the total sampling depth in different habitats and climate types. Sites where both substrate and cover were sampled; total n = 1,290 sites. Source data are provided as a Source Data file.

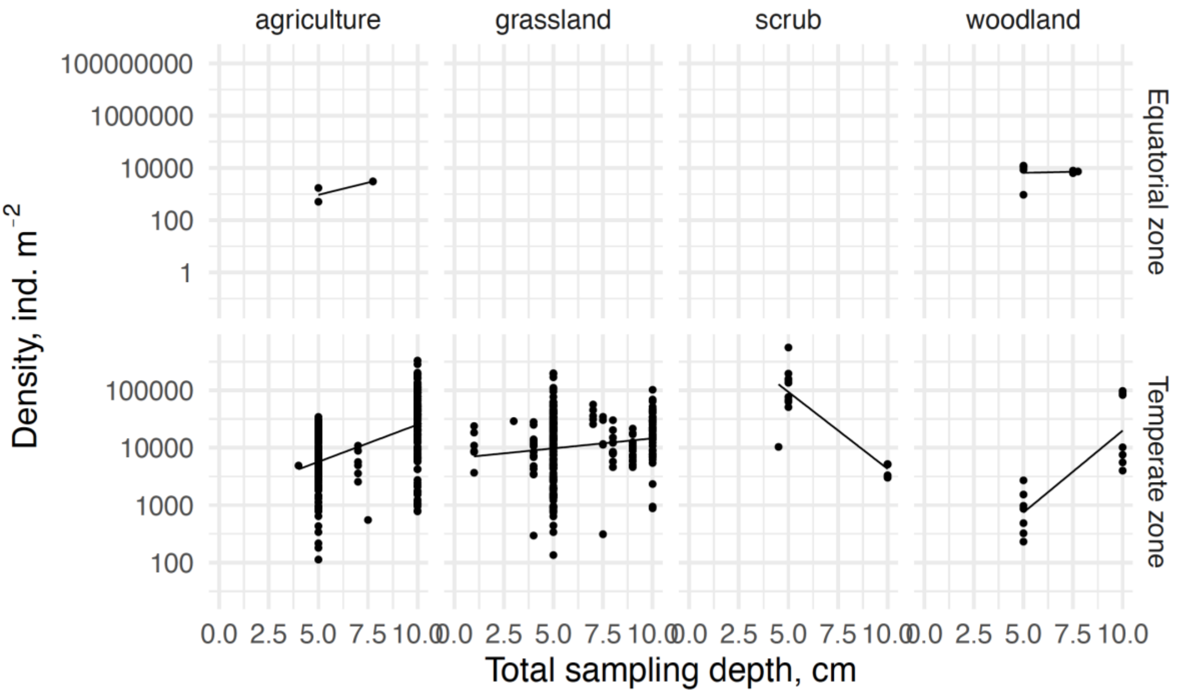

Supplementary Fig. 14 | Density of Collembola depending on the total sampling depth in different habitats and climate types. Sites where only substrate (soil) was sampled; total n = 908 sites. Source data are provided as a Source Data file.

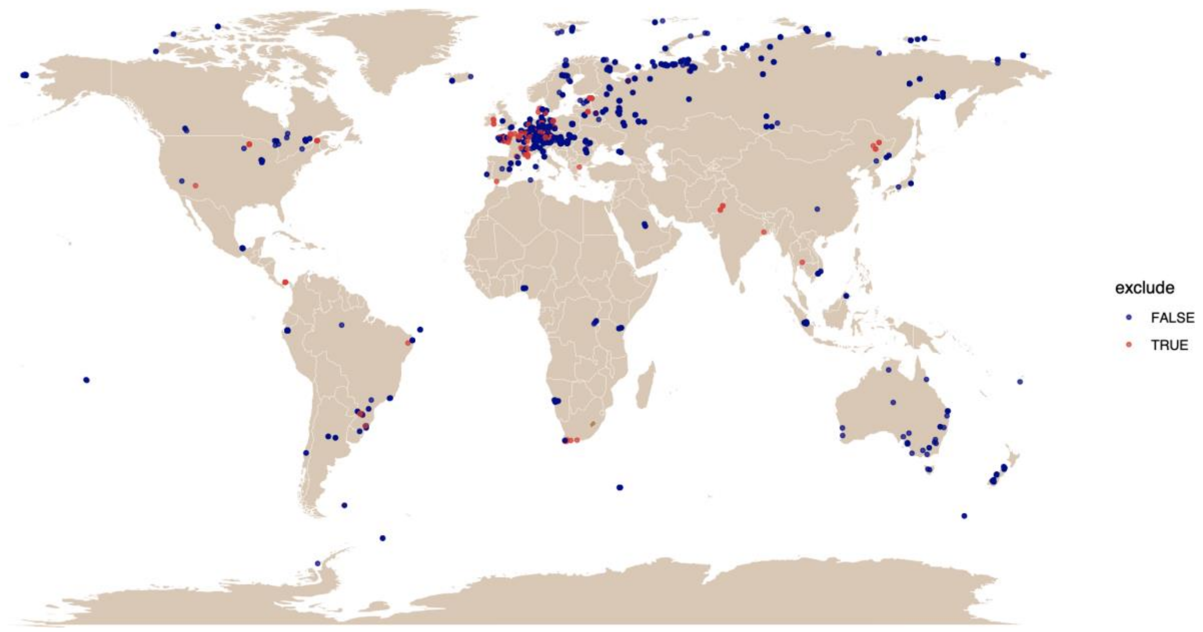

Supplementary Fig. 15 | Sampling sites that were excluded from the analysis of springtail density. Sites were excluded due to an absence of density estimation and potentially underestimated density (marked with red colour; total n = 2,471 sites). Exclusion was made both on expert evaluation and data exploration. Source data are provided as a Source Data file.

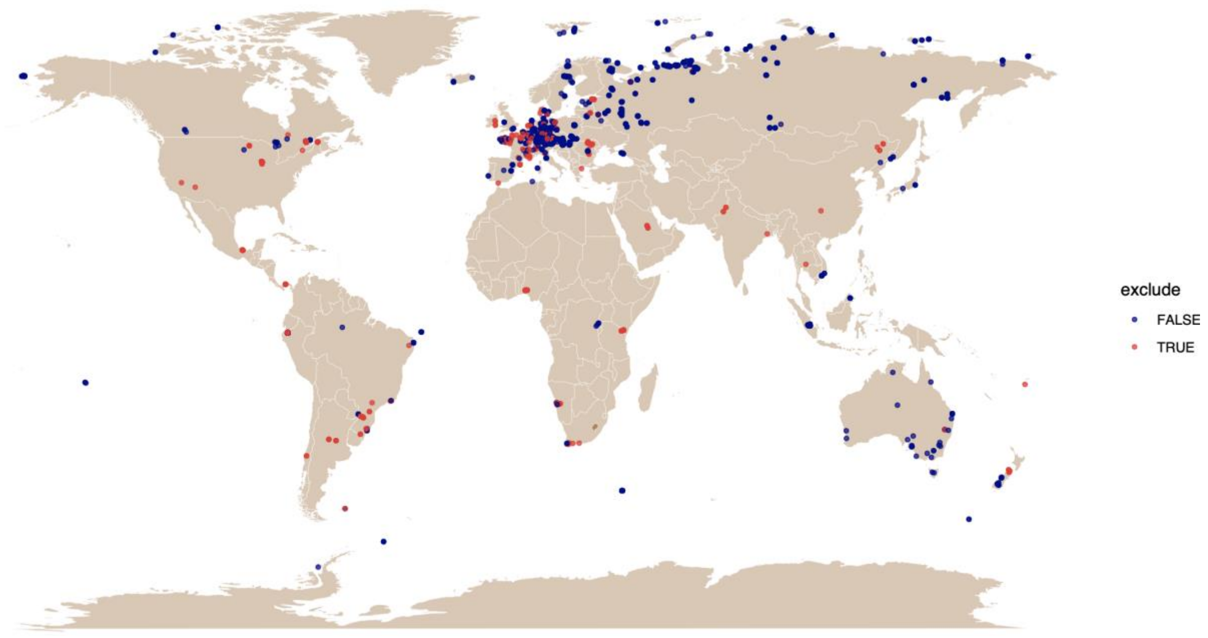

Supplementary Fig. 16 | Sampling sites that were excluded from the analysis of springtail biomass and metabolism. Sites were excluded due to an absence of density estimation and potentially underestimated density (marked with red color; total n = 2,471 sites). Exclusion was made both on expert evaluation and data exploration. Source data are provided as a Source Data file.

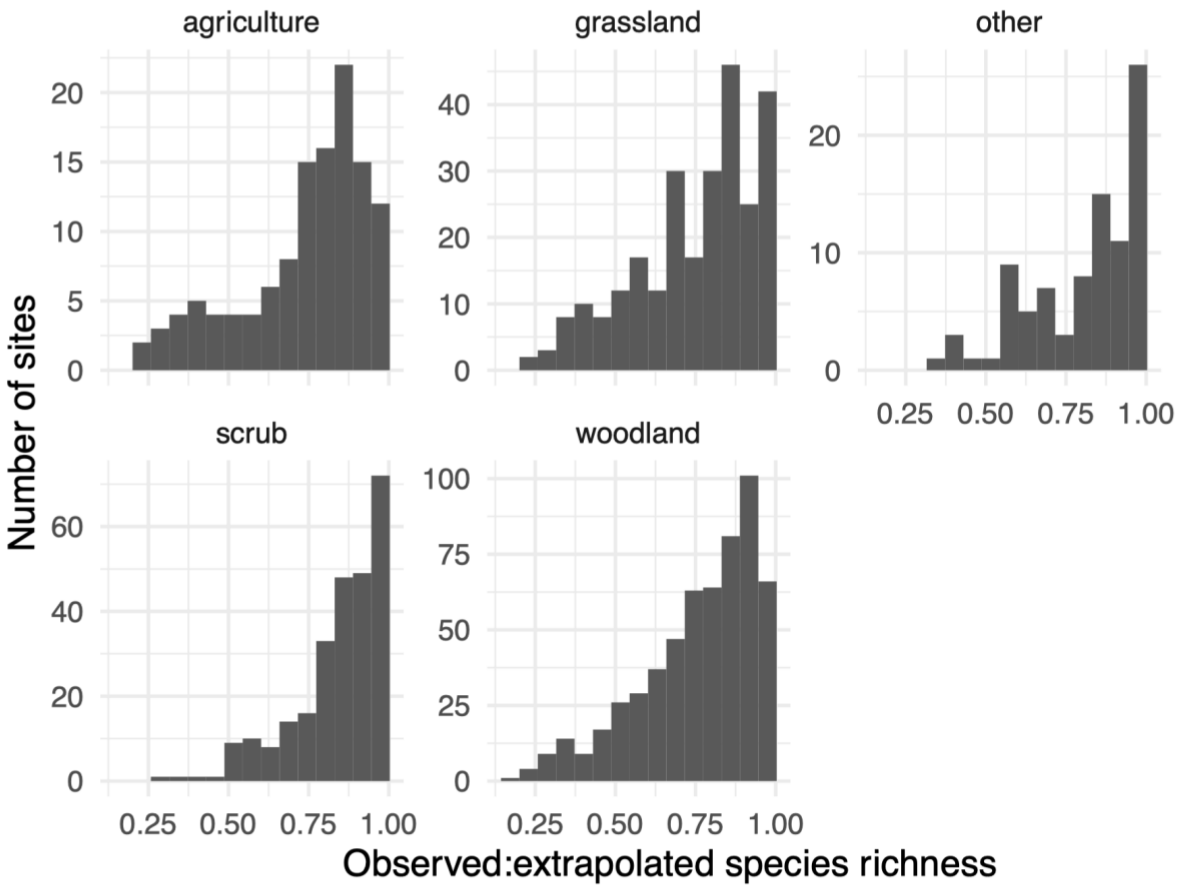

Supplementary Fig. 17 | Distribution of ratios of observed to extrapolated species richness in different habitats. Only datasets where sample-level data were recorded (agriculture n = 141, grassland n = 334, other n = 93, scrub n = 292, woodland n = 601). Source data are provided as a Source Data file.

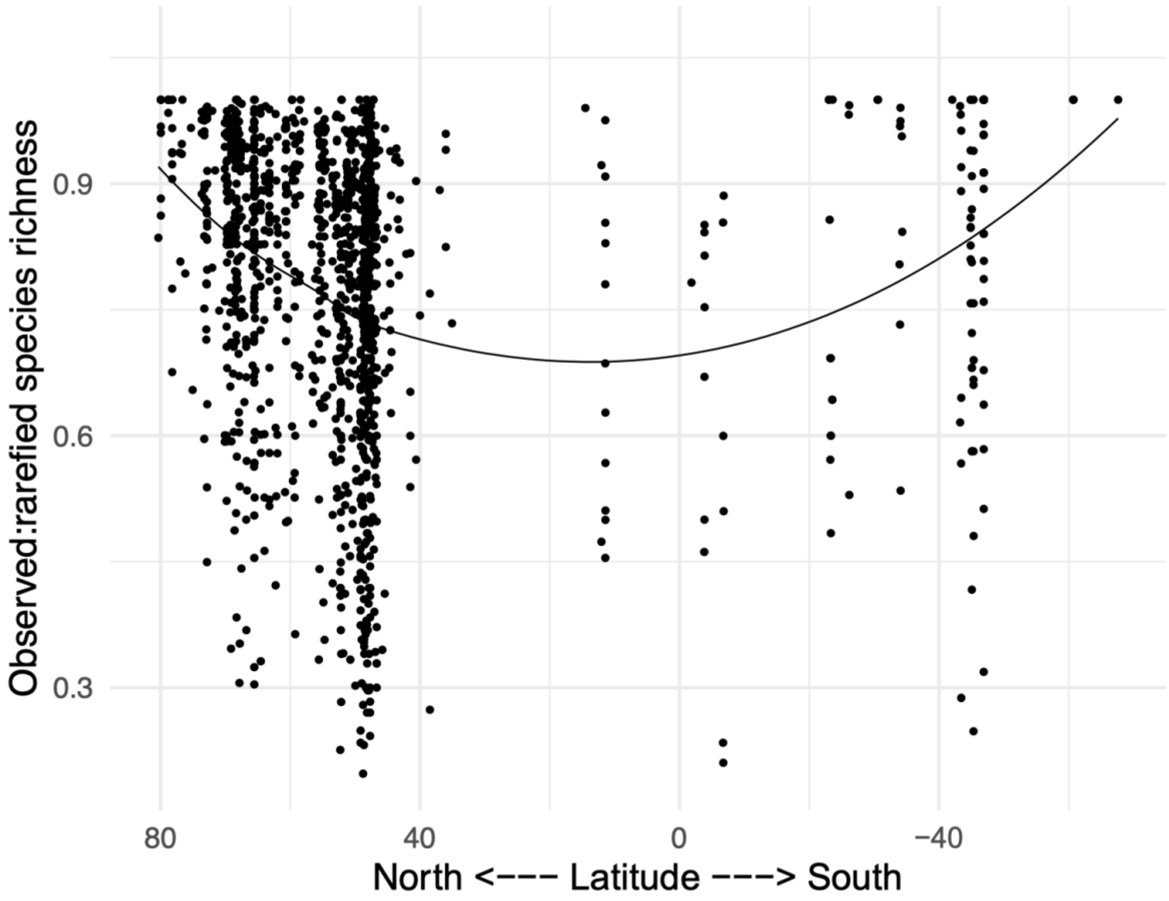

Supplementary Fig. 18 | Ratio of observed to extrapolated species richness across latitudes. Non-linear smoother is shown to illustrate trends. Total n = 1,461 sites. Source data are provided as a Source Data file.

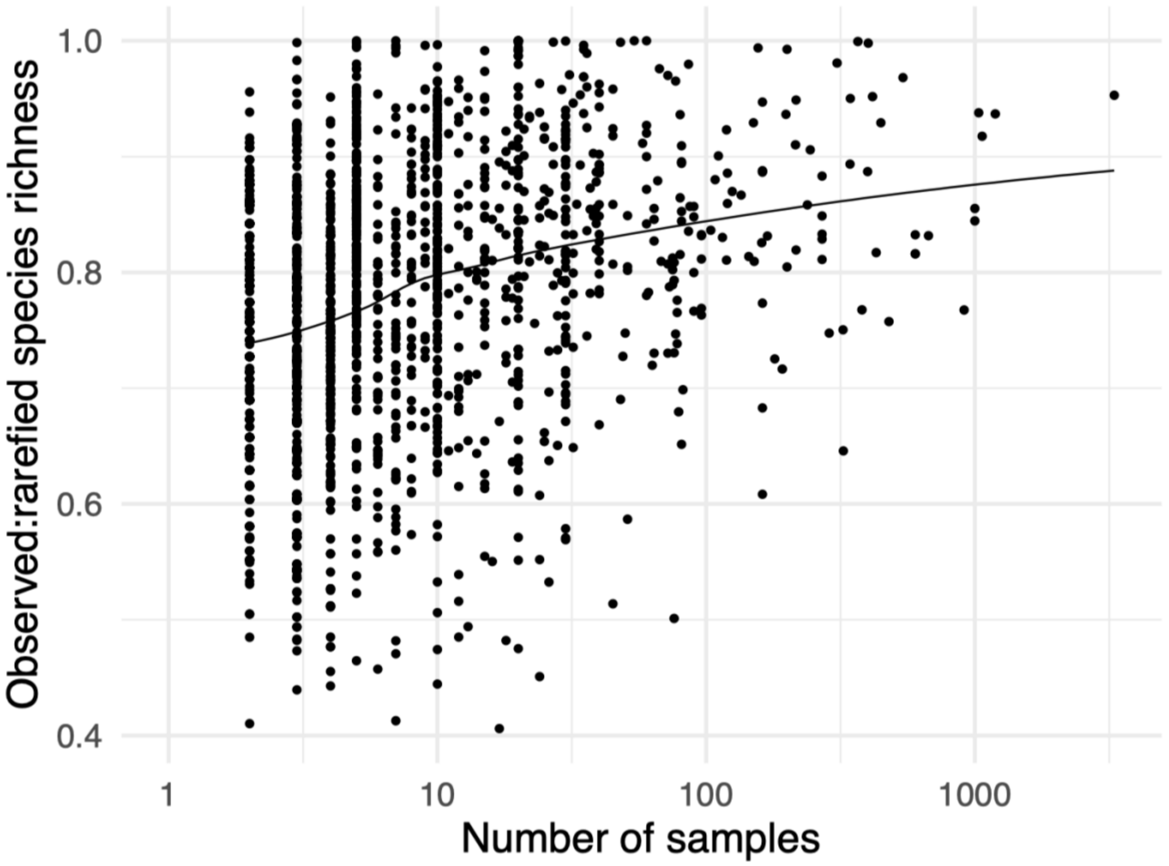

Supplementary Fig. 19 | Ratio of observed to extrapolated species richness depending on the number of samples. Non-linear smoother is shown to illustrate trends. Total n = 1,461 sites. Source data are provided as a Source Data file.

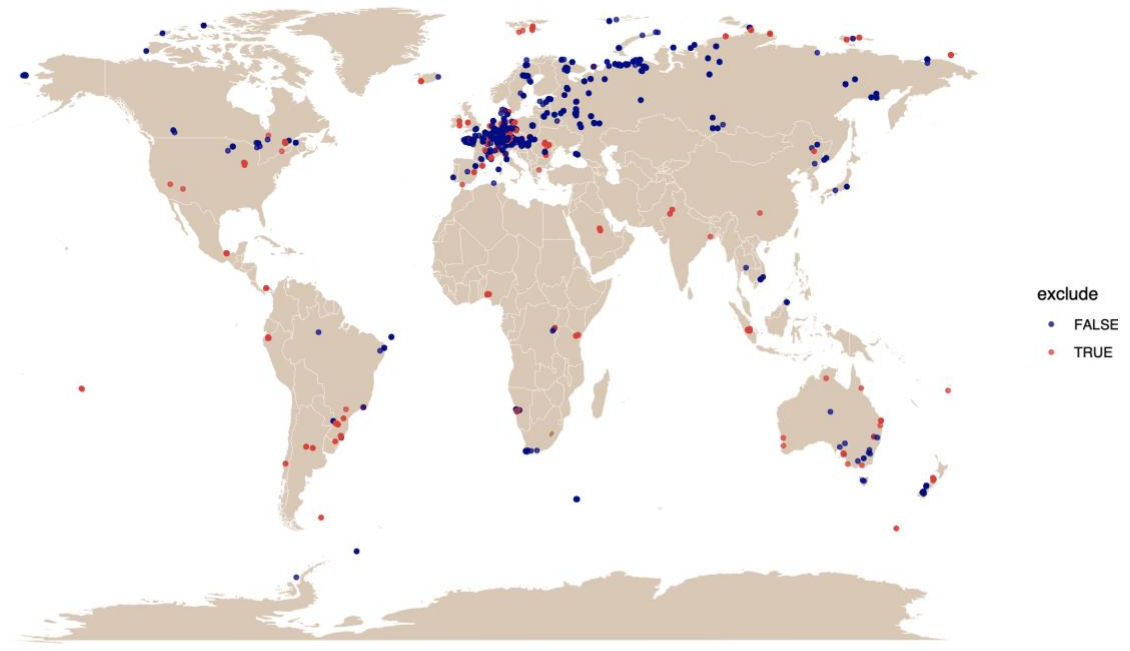

Supplementary Fig. 20 | Sampling sites that were excluded from the analysis of springtail species richness. Sites were excluded due to an absence of species-level diversity estimation and potentially underestimated diversity (marked with red colour; total n = 2,471 sites). Exclusion was made based both on expert evaluation and data exploration. Source data are provided as a Source Data file.
