# Supplementary material for: Globally invariant metabolism but density-diversity mismatch in springtails
Source: Nat Commun. 2023 Feb 7;14:674. doi: 10.1038/s41467-023-36216-6 (PMC9905565; doi:10.1038/s41467-023-36216-6)
Supplement: Supplementary file 3 — Reporting Summary [file 41467_2023_36216_MOESM3_ESM.pdf]

nature portfolio

Corresponding author(s): Anton M. Potapov

Last updated by author(s): Dec 23, 2022

Reporting Summary

Nature Portfolio wishes to improve the reproducibility of the work that we publish. This form provides structure for consistency and transparency in reporting. For further information on Nature Portfolio policies, see our Editorial Policies and the Editorial Policy Checklist.

Statistics

For all statistical analyses, confirm that the following items are present in the figure legend, table legend, main text, or Methods section.

n/a	Confirmed	
☐	☒	The exact sample size (*n*) for each experimental group/condition, given as a discrete number and unit of measurement
☐	☒	A statement on whether measurements were taken from distinct samples or whether the same sample was measured repeatedly
☐	☒	The statistical test(s) used AND whether they are one- or two-sided<br>*Only common tests should be described solely by name; describe more complex techniques in the Methods section.*
☐	☒	A description of all covariates tested
☐	☒	A description of any assumptions or corrections, such as tests of normality and adjustment for multiple comparisons
☐	☒	A full description of the statistical parameters including central tendency (e.g. means) or other basic estimates (e.g. regression coefficient) AND variation (e.g. standard deviation) or associated estimates of uncertainty (e.g. confidence intervals)
☐	☒	For null hypothesis testing, the test statistic (e.g. *F*, *t*, *r*) with confidence intervals, effect sizes, degrees of freedom and *P* value noted<br>*Give P values as exact values whenever suitable.*
☒	☐	For Bayesian analysis, information on the choice of priors and Markov chain Monte Carlo settings
☒	☐	For hierarchical and complex designs, identification of the appropriate level for tests and full reporting of outcomes
☒	☐	Estimates of effect sizes (e.g. Cohen's *d*, Pearson's *r*), indicating how they were calculated

Our web collection on statistics for biologists contains articles on many of the points above.

Software and code

Policy information about availability of computer code

Data collection	Excel of Mac v 16.55 (Microsoft); Open Refine v 3.4.1. (open source, community supported)
Data analysis	Custom code was generated to calculate basic estimators, path analysis and display the data in R version 4.0.2 (open source, R Core Team) with RStudio interface v. 1.4.1103 (RStudio, PBC). We used the following R packages: tidyverse v1.3.1, plyr v1.8.7, outliers v0.15, naniar v0.6.1, EnvStats v2.7.0, lavaan v0.6-12, reshape2 v1.4.4, reshape 0.8.8, taxize v0.9.100, scales v1.2.0, mapsv3.4.0, stringr v1.4.0. Custom code was generated for geospatial modeling in Python version: 3.6.5 (open source, Python software foundation). All Earth Engine (Google) code was written in Javascript. We used the following Python packages beyond the standard library: numpy v1.19.5, pandas v1.3.0, sklearn (scikit-learn) v0.24.2, scipy v1.7.0, tqdm v4.61.2, ee v0.1.328. Programming code for the path analysis and the geospatial modelling are available under CC-BY 4.0 from Figshare: https://doi.org/10.6084/m9.figshare.16850419.

For manuscripts utilizing custom algorithms or software that are central to the research but not yet described in published literature, software must be made available to editors and reviewers. We strongly encourage code deposition in a community repository (e.g. GitHub). See the Nature Portfolio guidelines for submitting code & software for further information.

Data

Policy information about availability of data

All manuscripts must include a data availability statement. This statement should provide the following information, where applicable:

- Accession codes, unique identifiers, or web links for publicly available datasets
- A description of any restrictions on data availability
- For clinical datasets or third party data, please ensure that the statement adheres to our policy

The data that support the findings of this study are available under CC-BY 4.0 license from Figshare: https://doi.org/10.6084/m9.figshare.16850419; high-resolution maps can be assessed at https://doi.org/10.6084/m9.figshare.16850446. We partly acquired body length data of Collembola from BETSI: https://portail.betsi.cnrs.fr/node/2

Human research participants

Policy information about studies involving human research participants and Sex and Gender in Research.

Reporting on sex and gender	The study does not involve any human research participants. We did not select or report springtail data based on sex since it was out of the scope of this study
Population characteristics	See above
Recruitment	We used all available data
Ethics oversight	Not applicable

Note that full information on the approval of the study protocol must also be provided in the manuscript.

Field-specific reporting

Please select the one below that is the best fit for your research. If you are not sure, read the appropriate sections before making your selection.

☐ Life sciences ☐ Behavioural & social sciences ☒ Ecological, evolutionary & environmental sciences

For a reference copy of the document with all sections, see nature.com/documents/nr-reporting-summary-flat.pdf

Ecological, evolutionary & environmental sciences study design

All studies must disclose on these points even when the disclosure is negative.

Study description	This study used abundances and taxonomic identifications from 2,470 local springtail communities to assess the latitudinal gradients and the global distribution drivers and patterns of density, species richness, biomass and community metabolism of soil springtails. We used globally available environmental variables in a factorial analysis (spatial extrapolation with random forest) and in a factorial-hierarchical way (structural-equation modelling).
Research sample	Each sample in the analysis is represented by a sampling site where density and/or species richness of springtail communities were described. Each site-level average estimate is characterized by several soil samples (6 is the median) from which springtails were extracted. Data were primarily collected from individual archives of contributing co-authors and complemented with published literature and existing databases (Edaphobase). The initial data collection was unselective.
Sampling strategy	Animals were collected from soil samples using dry extraction methods. The nuanced methodology varied from dataset to dataset. No sample size calculation was performed; the sample size was deemed to have sufficient spatial coverage compared to that used in previous global soil biogeographic studies. The representativeness of the dataset was assessed using multivariate analysis across the 73 environmental layers included in the geospatial modeling.
Data collection	Sample- and site-level data were collected from published literature, unpublished personal datasets and Edaphobase. The initial collection of data was done by all co-authors, the compilation of data was done by the corresponding author.
Timing and spatial scale	Samples were collected worldwide with sampling dates ranging from 1970 to 2019. The data represents most of the seasons and years within this period. Data selection process did not involve temporal aspect.
Data exclusions	Data that falls out of the scope of the study (e.g. beyond soil and area-based collection methods) were excluded. Unreliable estimations were excluded using technical filtering and expert quality check (see Extended Data Fig. 1 for more details).
Reproducibility	Both the data and the programming code are available from Figshare: https://doi.org/10.6084/m9.figshare.16850419 allowing for a full reproduction of the analyses. However, the results might be slightly different due to bootstrapping approach used in both path analysis and geospatial modelling.

Randomization	Data were grouped by IPBES geographical regions to account for the spatial clustering of the data in Europe. The grouping was used to introduce bootstrapping approach in the analysis.
Blinding	No treatments groups were assigned in the study, as we performed an observational and exploratory geospatial mapping project.

Did the study involve field work? ☐ Yes ☒ No

Reporting for specific materials, systems and methods

We require information from authors about some types of materials, experimental systems and methods used in many studies. Here, indicate whether each material, system or method listed is relevant to your study. If you are not sure if a list item applies to your research, read the appropriate section before selecting a response.

Materials & experimental systems

n/a	Involved in the study
☒	☐ Antibodies
☒	☐ Eukaryotic cell lines
☒	☐ Palaeontology and archaeology
☐	☒ Animals and other organisms
☒	☐ Clinical data
☒	☐ Dual use research of concern

Methods

n/a	Involved in the study
☒	☐ ChIP-seq
☒	☐ Flow cytometry
☒	☐ MRI-based neuroimaging

Animals and other research organisms

Policy information about studies involving animals; ARRIVE guidelines recommended for reporting animal research, and Sex and Gender in Research

Laboratory animals	Not applicable
Wild animals	The data used in this study is based on field-sampled springtails (small-sized invertebrates). Due to specifics of the collection methods, animals are killed to acquire data.
Reporting on sex	Information on sex has not been collected and considered in the study, being out of the scope.
Field-collected samples	Springtails are commonly stored in ethanol or on microscopic slides. Since the analysis is based on a compilation of studies, different storage treatments might have been applied in each individual source study.
Ethics oversight	Regulations for an ethical approvement for springtail studies are not present.

Note that full information on the approval of the study protocol must also be provided in the manuscript.
